# Genetic Signatures for Enhanced Olfaction in the African Mole-Rats

**DOI:** 10.1371/journal.pone.0093336

**Published:** 2014-04-03

**Authors:** Sofia Stathopoulos, Jacqueline M. Bishop, Colleen O’Ryan

**Affiliations:** 1 Department of Molecular and Cell Biology, University of Cape Town, Cape Town, Western Cape, South Africa; 2 Department of Biological Sciences, University of Cape Town, Cape Town, Western Cape, South Africa; Duke University, United States of America

## Abstract

The Olfactory Receptor (OR) superfamily, the largest in the vertebrate genome, is responsible for vertebrate olfaction and is traditionally subdivided into 17 OR families. Recent studies characterising whole-OR subgenomes revealed a ‘birth and death’ model of evolution for a range of species, however little is known about fine-scale evolutionary dynamics within single-OR families. This study reports the first assessment of fine-scale OR evolution and variation in African mole-rats (Bathyergidae), a family of subterranean rodents endemic to sub-Saharan Africa. Because of the selective pressures of life underground, enhanced olfaction is proposed to be fundamental to the evolutionary success of the Bathyergidae, resulting in a highly diversified OR gene-repertoire. Using a PCR-sequencing approach, we analysed variation in the OR7 family across 14 extant bathyergid species, which revealed enhanced levels of functional polymorphisms concentrated across the receptors’ ligand-binding region. We propose that mole-rats are able to recognise a broad range of odorants and that this diversity is reflected throughout their OR7 gene repertoire. Using both classic tests and tree-based methods to test for signals of selection, we investigate evolutionary forces across the mole-rat OR7 gene tree. Four well-supported clades emerged in the OR phylogeny, with varying signals of selection; from neutrality to positive and purifying selection. Bathyergid life-history traits and environmental niche-specialisation are explored as possible drivers of adaptive OR evolution, emerging as non-exclusive contributors to the positive selection observed at OR7 genes. Our results reveal unexpected complexity of evolutionary mechanisms acting within a single OR family, providing insightful perspectives into OR evolutionary dynamics.

## Introduction

Highly developed olfaction and odour discrimination underpin a number of fitness-related behaviours in mammals, from foraging and predator avoidance, to individual recognition, mate choice and maternal care [1]–[3]. In vertebrates, odour molecules are detected by seven trans-membrane G-protein-coupled receptors (7-TM GPCRs) encoded by the olfactory receptor (OR) gene family - the largest in the vertebrate genome [4]. From available genome data, it is clear that the extent of the vertebrate OR repertoire varies considerably, ranging from ∼100 genes in fish [5], to 400–1000 ORs in tetrapods (from 388 functional ORs in humans to 1259 functional genes in rats; [5]–[7]), where the expansion of OR gene repertoires is thought to reflect the shift from aquatic to terrestrial environments in the Middle Devonian, some 395 MYA [8]. As with the evolution of most multi-gene families, dynamic and rapid evolution via the birth-and-death model has been proposed for the OR gene family. Here, new OR genes arise through duplication and then either diversify in function in response to selection, lose function via pseudogenization, or are lost from the genome [9]–[11]. Thus, the extent of any OR repertoire (i.e. number of genes and the diversity among these genes) depends on diverse evolutionary forces, as well as the extent of duplication and inactivation events that characterise the evolution of a species’ genome [11].

Vertebrate ORs are predominantly expressed in the sensory neurons of the main olfactory epithelium (MOE) [12]; further evidence also supports their expression in the rodent vomeronasal organ (VNO) and septal organ of Masera [13].

Genetic variation within OR genes is concentrated in the ligand-binding pockets of the receptors, spanning trans-membrane domains 2–7 (TM 2–7) [12], [14], [15]. High levels of polymorphism in this region are associated with the recognition of a wide range of chemicals, including both odorants and semiochemicals [16]–[18]. While the overall structure of ORs is maintained by strong purifying selection, a signal of positive selection in the ligand-binding region is reported in a diverse range of species, from fish to rodents [19]–[21]. This is consistent with the evolutionary pressure to generate and maintain adaptive binding properties at ORs, for the recognition of ecologically important odorants across species and habitats [22].

Olfactory acuity in vertebrates is commonly measured using the number of ‘functional’ OR genes in a species genome, together with the ratio of functional OR genes: pseudogenes [18], [23], [24]. Functional OR gene number is thought to be proportional to the range of scents that can be detected and discriminated between [17], [18]. On the other hand, the ratio of OR genes:pseudogenes depends on the evolutionary forces that have shaped the OR repertoire of a species. Accordingly, these two measures vary across species, as a result of both lineage age and the selective environment in which they have evolved. For example, a number of extant rodent species, known to rely on highly developed olfaction for fitness-related tasks, have a large proportion of functional OR genes in their repertoires. In contrast, in species where olfaction has regressed, there is a higher fraction of OR pseudogenes. For example, in primates the evolution of full trichromatic vision is proposed to have influenced loss of OR diversity [25] (but see [26]).

Increasing evidence supports a role for ecological niche adaptations in the evolution of the vertebrate OR repertoire. A recent comparative survey of mammalian OR subfamily diversity, proposed a significant role for ecological niches in the evolution of OR functional diversity [22]. Similarly, the loss of OR functionality in cetaceans appears directly related to the evolution of an aquatic lifestyle [27]. Noteworthy, is the higher proportion of functional ORs reported in baleen whales (Mysticeti), which have a complex olfactory bulb, in comparison to toothed whales (Odontoceti), implying greater olfactory ability in mysticetes. This increased olfactory sensitivity is hypothesised to enable mysticetes to orientate more successfully toward aggregations of their dominant food source, krill [28]. Likewise, elapid snakes, viviparous species that have recently adapted to a marine lifestyle (Subfamily Hydrophiinae, ∼8 MYA; [29]), have also experienced extensive OR pseudogenisation in comparison to both oviparous aquatic snake species, which still require land-based nests for their eggs, and fully terrestrial species [30]. In birds, a larger OR repertoire is found in a number of nocturnal species, that are known to rely on olfactory cues, as compared to their closest diurnal relatives [31]. Thus, the physical environment clearly influences functional diversification and size of this multi-gene family [27], [30], [32].

Here we explore OR diversity and evolution within a single OR family, namely OR7, in the African mole-rats. These burrowing rodents of the family Bathyergidae are endemic to sub-Saharan Africa, and most notable for their broad range of social strategies [33]. Whilst they do disperse above-ground, mole-rats essentially live permanently underground and have evolved an array of morphological, physiological and behavioural adaptations [33], [34]. All species are poorly equipped for utilisation of the visual field [35] and exhibit little neuro-anatomical or molecular evidence of adaptation for low-light vision [36]–[38]. Whilst light/dark discrimination has been reported, the bathyergid central visual system is significantly reduced [37], [39] and, in the absence of visual cues, all species exhibit enhanced olfactory sensitivity [33], [36]. Olfactory cues direct mole-rats digging towards food resources, thus minimising the energy investment necessary for successful foraging [40], [41]. For example, naked mole-rats, *Heterocephalus glaber*, recruit colony members to food sources by laying down odour trails [42], and similarly use olfactory cues during colony interactions [43]–[46]. Furthermore, complex scent marking rituals are used in common nesting and latrine areas within the extensive burrow systems of all the social mole-rats [33], [47]. This chemo-communication in naked mole-rats is perhaps surprising, given that they lack a functional vomeronasal organ (VNO) [48]. Thus, pheromonal communication in naked mole-rats may be mediated by the MOE in a similar manner to that hypothesised for humans [49]. Other examples of chemo-communication in bathyergids are reported in species of the social genus *Cryptomys,* where individuals are able to discriminate between kinspecific and heterospecific odours using a proposed “self-referent matching” mechanism [50]–[52]; this information is used to both reinforce individual and group recognition rituals and to limit incestuous mating [53].

Given the socio-ecological significance of odour discrimination in the Bathyergidae, we examined OR7 diversity across all genera of extant mole-rats and present the first assessment of OR gene diversity and evolution in a subterranean mammal. Useing PCR and sequencing methods, we characterise representative OR7 diversity across 14 bathyergid species and classify bathyergid OR genes, based on phylogenetic relationships together with a range of published OR subgenomes. We hypothesize that well-developed olfaction in Bathyergidae is the result of an expansion within the OR multi-gene family, resulting in increased divergence among OR7 genes. We also test whether patterns of OR7 variation in the amino acids involved in ligand-binding, are consistent with a scenario of adaptive functional variability across the Bathyergidae. In this context, we use phylogenetic-based methods to test whether adaptive evolution has operated differentially across bathyergid OR7 clades. Finally, we investigate a role for sociality and environmental niche specialisation in determining OR7 gene diversity in mole-rats and interpret our results within the framework of Nei’s ‘birth-and-death’ model of evolution for multi-gene families [9].

## Results

### Olfactory Receptor Diversity in African Mole-rats

The Bathy-OR1/Bathy-OR2 primer pair were designed in this study and yielded unambiguous amplification of OR7 loci in all 14 African mole-rat species This produced a final alignment of 178 unique OR7 sequences (GenBank accession numbers KF453235–KF453412), and a BLAST search confirmed the sequence identity as OR7 genes for all sequences in the dataset. A ‘conserved domains’ search revealed the presence of typical GPCRs features in all sequences [54], whilst known OR motifs were confirmed by eye from the amino acid alignment [23], [55].

Consistent with published studies, mole-rat OR sequences were considered to be pseudogenes if they had mutations that disrupted the 7TM receptor structure; these mutations included stop codons and frameshift mutations [8], [31], [56]. Using these criteria, 97 of the 178 bathyergid OR sequences were classified as pseudogenes. However, this may be a potential underestimation of the number of pseudogenes because of additional mutations outside the amplified region (TM 2–7), or mutations in promoter regions that were not amplied [25], [57].

After allelic variants were merged, 119 unique OR7 genes were identified from the original pool of 178 OR7 gene candidates, including 51 putatively functional ORs and 68 OR pseudogenes. Interestingly, alleles of the same OR7 gene (as well as identical alleles) were identified across a number of mole-rat species and tentatively supports the idea that OR7 diversification may have preceded speciation in Bathyergidae.

**Figure 1:**
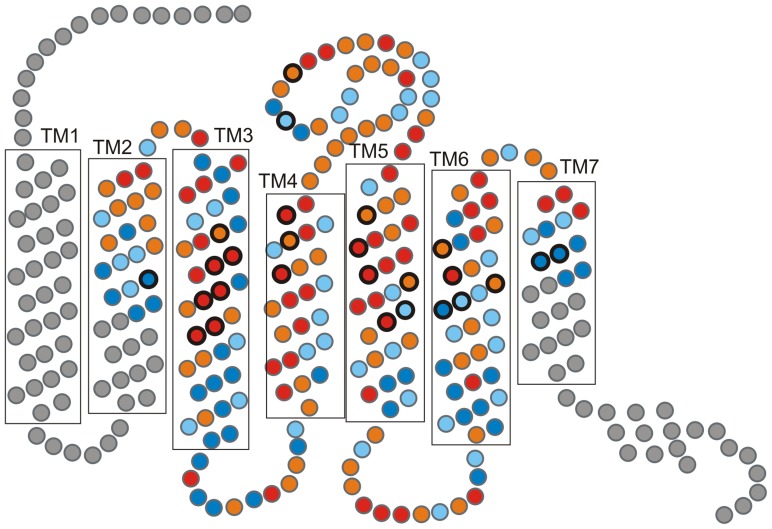
Functional variability across mole-rat OR7 receptors (redrawn from [15]). Functional variation is colour coded based on the number of different amino-acids presents at each position: red – highly variable (≥5); orange – variable (3–4); light blue – conserved (2); dark blue – highly conserved (1). Amino-acid positions involved odorant-binding are circled in black [14], [15]; these are predominantly variable in our dataset, as expected. Abbreviations stand for the following: TM trans-membrane domain, EC extra-cellular and IC intracellular domain.

The distribution of amino acid diversity across Bathyergidae OR7 genes was assessed based on Katada et al.’s molecular model of the mouse mOR-EG receptor [15]. The topological distribution of conserved and variable sites in mole-rat receptors is analogous to that of mOR-EG [15], with 73% of highly conserved residues shared, and 88% of variable residues occupying the same locations (Figure 1). High levels of both nucleotide and amino acid sequence polymorphism were detected in mole-rat OR7 sequences, and variability is concentrated in the region between TM3 and TM6, which corresponds to the predicted core of the ligand-binding pocket of ORs (Figure 1) [14], [15]. Interestingly, 19 of the 26 amino acid residues predicted to be involved in ligand-binding [14], [15], are variable in bathyergids. If residues in TM domains 2 and 7 are excluded, 83% of the alleged odorant-binding sites in mole-rat OR7 genes are polymorphic, consistent with a role in odorant recognition [14].

In keeping with other published studies, recombination is not a significant mechanism for the generation of sequence variability across mole-rat OR7 loci [10]. Tests for linkage disequilibrium did not indicate significant pairwise associations between polymorphic sites (ZZ = 0.006). This is consistent with the widely accepted idea that variability across OR genes is predominantly the result of gene duplication events and nucleotide substitution driven by positive selection, rather than recombination [10]. This result means that recombinant PCR artefacts are unlikely to have obscured the signal in our dataset [58].

### Phylogenetic Relationships among Mole-rat OR7 Sequences

Phylogenetic reconstruction revealed four well-supported clades of closely-related OR7 genes (bootstrap support ≥97%). The four clades were named clades A–D, and an isolated gene (BJ4_A12), that is a sister lineage to clades A and B, was also observed. Identical phylogenetic topology was recovered when only a single representative sequence for each putative OR7 gene was analysed. The numbers and ratios of functional OR7 genes and pseudogenes across clades A–D are reported in Table S1.

The four clades do not cluster in a species-specific way. Instead, sequences in each clade were found to share functional motifs across the ligand-binding sites. Of the 23 amino-acid positions involved in odorant-binding across TM3-6 [14], [15], only three were found to be conserved across all clades, whilst the remaining 20 sites displayed clade-specific motifs. This is consistent with OR7 genes in each clade having different binding properties. Furthermore, there is a striking prevalence of hydrophobic amino-acids (92% of all amino-acids involved in odorant-binding) across the putative ligand-binding domain of OR7 genes in all clades. This result supports Katada et al.’s hypothesis [15], that the interaction between ORs and odorant ligands occur primarily via hydrophobic and van der Waals interactions [59].

### Classification and Evolution of Mole-rat OR7 Genes

Using genetic similarity criteria, mammalian OR genes are subdivided into Class I and Class II genes [60], [61], and these classes are further partitioned into 17 families. There are four Class I families, families 51, 52, 55 and 56, and 13 Class II families, families 1 to 13 [61], [62]. Although the differential functions of these families and the range of odorants they can recognise is poorly understood [5], it has been mooted that each family might detect a particular class of odorant molecules [63].

**Figure 2:**
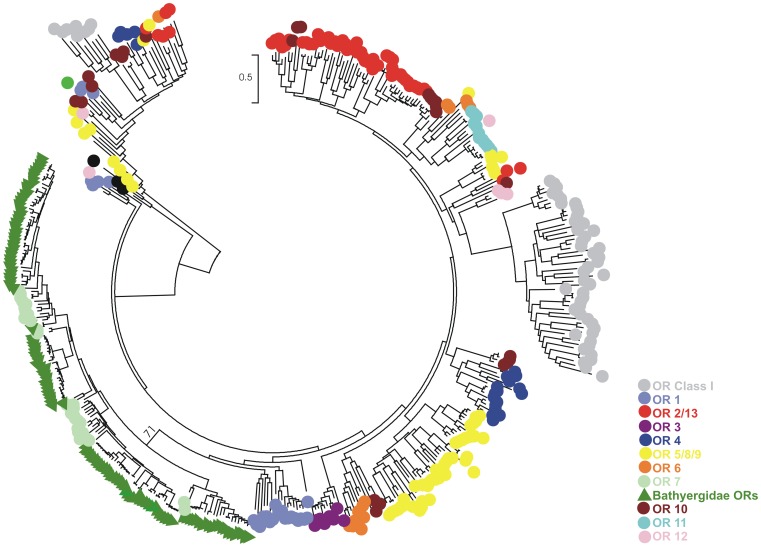
Mammalian OR family structure. Maximum likelihood tree obtained with Tamura-Nei substitution model (1000 bootstrap) using representative sequences of all OR Families from the available Mammalian database [22], together with the Bathyergidae OR genes characterised in this study. OR families are colour-coded as reported on the right. All Bathyergidae ORs appear to cluster together with mammalian Family 7 OR genes (indicated in green, together with the bootstrap support value for that branch).

In order to identify the OR genes amplified in our study, we inferred phylogenetic relationships between mole-rat OR genes and representative OR sequences from the entire OR repertoires of 18 different mammalian species [22]. The resulting phylogeny reveals strong support for Bathyergidae OR genes clustering together with Family 7 OR (OR7) genes from a number of mammalian species (Figure 2).

Family 7 OR genes represent a polyphyletic family of Class II OR genes in mammals, and are classified as part of the larger grouping of families 1/3/7 [22]. However, OR genes from families 1 and 3 appear to group independently from family 7, in strongly supported clades in our tree (Figure 2), and are more distantly related to the mole-rat OR genes characterized in this study.

The evolution of OR7 Bathyergidae genes was inferred by phylogenetic analyses of all the available mammalian OR7 sequences from Hayden et al.’s dataset [22]. Again, African mole-rat OR7 genes clustered into four strongly supported clades, which correspond to clades A–D in the Bathyergidae phylogenetic tree (with the exception of two genes; Figure S2). Interestingly, clades A, B and D appear to be Bathyergidae-specific clades, whilst clade C included OR7 genes from other mammalian species. Other family-specific clades are highlighted in the tree by a colour-coded classification of mammalian OR7 genes (Figure S2).

### Signatures of Selection in the African Mole-rat OR7 Tree

**Figure 3:**
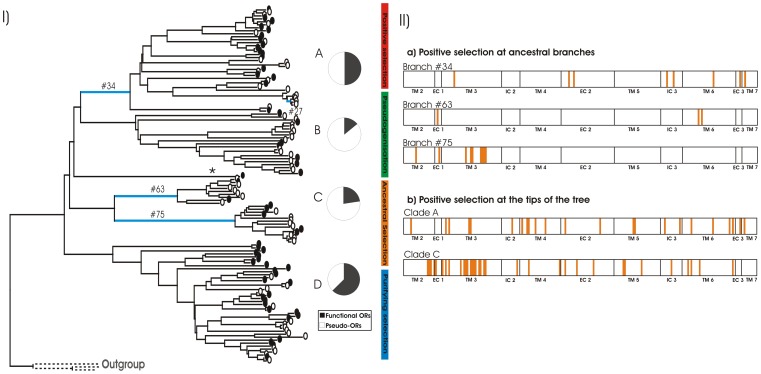
I Divergent evolutionary forces on the Bathyergidae OR7 gene tree. Simplified schematic view of the maximum likelihood tree (GTR, 1000 bootstrap) constructed using a single representative sequence for each putative Bathyergid OR gene; three rhodopsin-like GPCRs were used to root the tree (accession numbers NP_001287.2, NP_005292.2, NP_037014.2). Black filled circles at branch tips represent the putatively functional OR genes; empty circles represent OR pseudogenes. Pie charts represent the proportions of functional OR genes (black) and OR pseudogenes (white) in clades A–D; only one isolated gene falls out of these clades and is indicated with an asterisk. Positively selected lineages, according to branch-sites analysis, are coloured in blue; # branch numbers correspond to those assigned by CodeML [107], [108]. A summary of the selective forces acting on Bathyergidae OR7 gene family, based on ancestral and ongoing selection, as well as on the ratios and numbers of functional OR genes in each clade (Table S1), is represented in vertical colour bars. Figure 3.II Positively selected residues in Bathyergidae OR7 lineages. a) Results from the Bayes Empirical Bayes analysis reveal a prevalence of positively selected sites across the odorant-binding region of ORs (TM3-6); branch numbers match labelled branches in Figure 3.I. Amino-acid positions and location domains were assigned based on Katada et al.’s molecular model [15]. b) An analysis of ongoing selection on functional nucleotide alignments from clades A and C identifies a number of amino-acid positions characterised by dN/dS>1 (indicated in orange), whilst no such sites are found across clade D (clade B was excluded from the analysis for insufficient sample size).

Tests for differential positive selection across bathyergid OR7 clades revealed a number of evolutionary patterns. Likelihood ratio tests (LRT) for ongoing positive selection were performed on the functional OR7 genes from clades A, C and D, while clade B was excluded from this analysis due to insufficient sample size. The LRT results reveal significant positive selection for clade A only, although numerous codons were found to be evolving under positive selection in both clades A and C (Figure 3.II).

Notably, no amino-acid sites in clade D were characterised by dN/dS>1; instead, all sites within this clade were characterised by dN/dS ratios <1, which is consistent with purifying selective forces acting along the OR genes of this clade. A codon-based Z-test was then used and a strong signal of purifying selection was confirmed (p<0.0001).

To identify episodic events of adaptive evolution across specific Bathyergidae OR7 lineages, we used a branch-site test of positive selection across all branches of the African mole-rat OR gene tree. Six branches in the tree support a signal of positive selection in the corresponding lineages (p<0.05). However, when Q-values are taken into account, positive selection can only be inferred unequivocally for two branches (75 and 34, Q-value <0.001; Figure 3.I). The next two branches (# 27 and 63 Figure 3.I) are only mildly significant (Q-value = 0.13), whereas from the fifth branch the Q value jumps to 0.54. Results from a Bayes Empirical Bayes (BEB) [64], [65] analysis to identify which amino-acid sites are evolving under adaptive evolution, revealed that the number and location of positively selected sites vary among these lineages (reported in Figure 3.II and Table S2).

Divergent, lineage-specific evolutionary forces in mole-rat OR7 genes are revealed when considering signals of both current and episodic selection and the proportion of functional genes in of the four clades. Firstly, within clade A, significant adaptive selection was detected both at ancestral branches (branch #34, Figure 3.I) and at the tips of the tree. Clade A is also characterised by a relatively higher OR7 genes: pseudogenes ratio, when compared to other clades. Along branch #34, six of the eight amino-acid sites that were identified as evolving under positive selection, based on the BEB analysis, lie in TM3-6 region (Figure 3.II). This is suggestive of selection acting predominantly on the odorant-binding region in this gene lineage, presumably to generate novel binding properties. The second branch that carries a mild signal of positive selection in this clade (# 27 Figure 3.I), leads to a subset of *H. glaber* OR7 pseudogenes that have only one positively selected codon within TM2 (Table S2). In addition, only pseudogenes are present in the sub-clade derived from branch #27, further strengthening the idea that the mild signal of positive selection detected by the branch-site test may be a consequence of pseudogenisation, rather than adaptive evolution. Secondly, Clade B supports a small number of putatively functional OR7 genes, together with the highest proportion of pseudogenes in the dataset (86%, Figure 3.II). This is possibly indicative of the fact that ORs within this clade may be secondary for bathyergid olfaction and thus more susceptible to mutation, resulting in pseudogenes. Accordingly, the branch-site test of positive selection failed to detect any episodic events of positive selection across this clade. Thirdly, Clade C is characterised by a strong signal of positive selection on one ancestral branch (#63) and mild positive selection on the other ancestral branch (#75), with selection concentrated on the ligand-binding region of the genes (Figure 3 and Table S2). Although several codons at the tips of the tree are under positive selection, the LRT did not identify an unambiguous signal of selection. This, together with a relatively lower proportion of functional genes in this clade (23% functional OR7 genes in clade C *versus* 50% in clade A), is consistent with an ancestral pulse of adaptive evolution on clade C OR7 genes, perhaps indicating a phase when new OR functionalities were acquired within this gene lineage. Finally, strong purifying selection has maintained an unaltered pool of OR7 genes, over a long period of evolutionary time, in Clade D. Since the divergence of the major mole-rat genera *Bathyergus, Georychus, Cryptomys* and *Fukomys* ∼ 15–17MYA [66] (Figure S1) and throughout the phylogeny, no periods of adaptive evolution are detected in this clade. Interestingly, the highest proportion of functional OR7 genes (62.5%), as well as the greatest number of putatively functional genes, are found in clade D (Figure 3.II). These results are consistent with a scenario where odorant chemicals, that carry fundamental information for Bathyergidae fitness, are recognised by clade D ORs and are therefore actively maintained unchanged over time.

### The Roles of Sociality and Environment in Shaping OR7 Evolution

We tested whether episodic positive selection has acted differentially on OR7 genes across specific bathyergid lineages. The bathyergid OR7 gene phylogeny was partitioned between solitary and social species and explored with a branch-site test of positive selection (following Ramm et al. [67]). No significant correlation was found between social phenotypes and positive selection (LnL difference = 0, p = 1).

**Figure 4:**
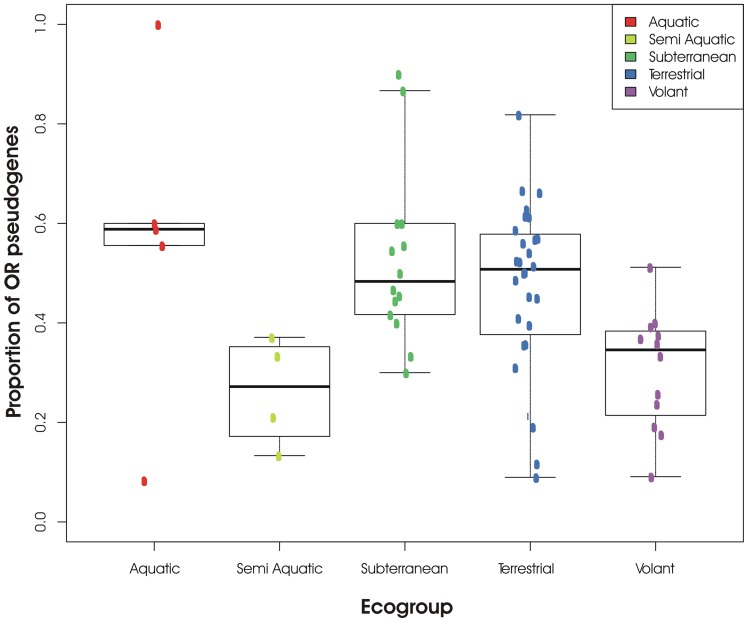
Proportions of OR 1/3/7 pseudogenes across Ecogroups. The mean percentage of pseudogenes and standard error are indicated for each Ecogroup.

The role of the environment in shaping OR7 diversity was also explored, by comparing OR genes of families 1/3/7 in mole-rats with a suite of mammalian species occupying the full spectrum of ecological habitats. Following Hayden et al. [22], the different proportions of OR 1/3/7 pseudogenes were calculated for each ecological habitat or ‘ecogroup’, and we introduced the mole-rat ‘Subterranean’ group to the analysis. Proportions of OR pseudogenes within ecogroups are reported in Figure 4.

A Wilcoxon rank-sum test was used to identify whether ecogroups differed significantly with respect to their proportions of (non)functional OR1/3/7. Significant differences were found between Subterranean and Semi-aquatic (p = 0.036), Subterranean and Volant (p = 0.011), and Terrestrial and Volant ecogroups (p = 0.014). A nearly significant value differentiates the Terrestrial from the Semi-Aquatic ecogroup (p = 0.06, Table 1). Thus, whilst the subterranean environment undoubtedly contributes to the evolution of the observed differences in OR 1/3/7 ratios across the broad range of mammals analysed, our data set is not significantly differentiated from the Terrestrial and Aquatic ecotypes.

**Table 1:** Pairwise comparisons between Ecogroups using Wilcoxon rank sum test (p-value adjustment method: BH).

	Aquatic	Semi-Aquatic	Subterranean	Terrestrial
**Semi-Aquatic**	0.317	–	–	–
**Subterranean**	0.505	**0.036** *	–	–
**Terrestrial**	0.483	0.06	0.81	–
**Volant**	0.127	0.518	**0.011** *	**0.014** *

*Ecogroups that differ significantly (p<0.05).

## Discussion

This study reports the first assessment of OR gene diversity in the African mole-rats and represents the first study of OR gene evolution in a subterranean mammal. Phylogenetic inference of a range of mammalian OR subgenomes identified the majority of sequences we recovered as belonging to the OR7 subfamily [22], [61]. We report evidence for a large number of functional polymorphisms that translate into diverse binding properties, as well as the presence of OR polymorphisms conserved across mole-rat species, indicating an ancient origin for some aspects of bathyergid OR7 diversification [68]. Our analysis of signatures of selection on mole-rat OR7 loci revealed evidence for clade-specific evolution of olfactory receptor genes. Our results are discussed in the context of the possible evolutionary drivers of OR7 diversification, and provide insight into the complex evolutionary history of a gene family that may be linked to individual fitness in this unusual mammalian lineage.

### Functional Variation across Bathyergid OR7 Genes

Four strongly supported OR7 lineages (clades A–D) were consistently recovered from all the OR phylogenies inferred in this study. Functional OR7 genes exhibited clade-specific motifs across the amino-acid sites involved in odorant-binding [14], [15], and we propose that ORs in each clade may have different binding properties. Although polymorphisms characterise these sites across clades, their chemical properties are similar, with a remarkable prevalence of hydrophobic residues across the putative ligand-binding OR domain. This finding is consistent with Katada et al.’s hypothesis [15], that the binding of odorant molecules in the odorant-binding pocket of ORs is mediated by hydrophobic interactions and van der Waals forces [59]. The OR binding-pocket spans TM3-6 and constitutes a binding environment that is broad i.e. able to recognise a range of odorants, but also selective for the shape, size and length of odorant ligands [15]. With the exception of a few known odorant-OR dyads, the functional characterisation of ORs and their respective ligands remains a major challenge [69]. Nevertheless, in humans single nucleotide polymorphisms in specific OR genes have been found to determine whether a specific odorant is detected or not [70]. Therefore, functional variability of the magnitude observed in our data set is consistent with a scenario where diverse binding properties have been selected for at OR7 loci.

Given the direct association between functional OR diversity and olfactory ability [17], [18], together with the role of olfaction in the socio-ecological success of bathyergids, we predicted that positive Darwinian selection has played a fundamental role in the evolution of variability at OR7 loci in the African mole-rats. Whilst we did find evidence for positive selection within our dataset, we also, somewhat unexpectedly, found strong evidence for divergent patterns of selection across the OR7 phylogenetic tree.

### A Role for Positive Selection in the Evolution of Bathyergid OR7 Genes

Positive selection is proposed to maintain functional variability at vertebrate OR loci, particularly in the ligand-binding region of ORs, while the overall receptor structure typical of GPCRs is thought to evolve under purifying selection [19], [20], [71]. Signatures of positive selection in clade A are similar to those reported for other vertebrate species [19]–[21], with selection acting predominantly on the ligand-binding domain of mole-rat OR7 genes. Similarly, two ancestral branches in clade C carry a signal of adaptive evolution across the receptors’ ligand-binding region and are likely the result of a historic pulse in positive selection on these loci. Based on such signals of positive selection, we suggest that functional variation at mole-rat OR7 loci was generated in response to selective pressures for enhanced sensitivity to the range of odorants recognised by mole-rats, and/or to optimise the recognition of crucial odorants. From this perspective, the detection of such odorant molecules may be directly related to fitness in mole-rats. Consistent with this scenario, adaptive evolution is likely an indicator of intra-specific competition for olfactorily-mediated resources [20]. Emes et al. [20] present the hypothesis that OR gene duplication and sequence diversification, driven by positive selection, are the result of intense competition between individuals, e.g. for food or predator avoidance. Unfortunately, there is limited information on specific ORs and their odorant ligands and it is therefore difficult to establish an explicit link between fitness and OR diversity at specific loci [63]. A theoretical association between OR variation and fitness is nonetheless indisputable, since ORs need to recognise odorants from an ever-changing environment, in a way that is perhaps comparable to the co-evolution of MHC receptors and the pathogen environment [71].

In this study we tested the possible roles of the subterranean environment and of the different levels of sociality in selecting for enhanced functional OR7 variation in mole-rats. The contribution of sociality (or solitariness), in shaping OR7 diversity, was explored using a tree-based method (following Ramm et al. [67]), but the analysis failed to indicate a significant correlation between the social phenotype of species and positive selection. While odour detection is of primary importance for mole-rats, the comparable degrees of selection detected in both social and solitary species may be the result of selection for functional diversification very early in the evolution of the bathyergid lineage. We integrated our dataset into a broad analysis of orthologous mammalian OR genes, to explore how environmental niche-specialisation may have influenced OR7 diversification in mole-rats. Proportions of (non)functional ORs across OR1/3/7 gene families, reveal that the Subterranean ecogroup differs significantly from the Volant and Semi-aquatic groups, but is not significantly different from the Terrestrial and Aquatic groups (Figure 4, Table 1). The lack of a significant difference between Terrestrial and Subterranean ecogroups may be biased by the heterogeneous taxonomic coverage in the two datasets analysed, together with the different ages of the taxa being compared. Species coverage in the Terrestrial ecogroup spans four superorders of mammals, with 28 species from more than 20 different families, and extremely variable lineage ages (e.g. Muridae 31 MY, Canidae 12 MY) [72], [73]. In contrast, only a single, relatively ancient mammalian family represents the Subterranean group (Bathyergidae 49 MY) [74], [75]. Ideally, a more balanced species coverage across ecogroups, considering only those taxa with similar ages, could be used to test more accurately for the role of environment. Because continuous ‘birth and death’ evolution theoretically leads to an increase of OR pseudogenes, which are essentially neutral [76], [77], older species may have accumulated a greater proportion of pseudogenes simply as a function of time. Even though OR pseudogenes will eventually become unidentifiable due to accumulated mutations, some ORs classified as ‘non-functional’ may still play a regulatory role in gene expression. Zhang et al. [78] report that 67% of human pseudogenes are in fact transcribed and this may explain the persistence of OR ‘pseudogenes’ in the genome over long periods of time. A further caveat, given our methodology, is that our data set is unlikely to be fully representative of the true pattern in the Bathyergidae, and analysis of the recently published naked mole-rat genome (http://naked-mole-rat.org) will provide valuable insight into this question in future studies. Nevertheless, a role for sociality and the environment in shaping and/or maintaining OR variation in mole-rats cannot be excluded. Undoubtedly, olfactory requirements will differ between solitary and social bathyergid species because of the fundamental differences in lifestyles. For example, social species require a mechanism to optimise kin recognition and use this behaviour to avoid incestuous matings and maintain colony cohesion [44], [53], [79]. The observed tendency of the subterranean environment to influence OR7 diversity, is only partly consistent with Hayden et al.’s conclusions [22] that natural selection, via niche-specific adaptation, shapes OR subgenomes. Nonetheless, it is reasonable to propose that the olfactory requirements of species that inhabit such diverse ecogroups are different and may be reflected in other OR gene families. The necessity to detect either airborne or water-soluble odorants is the most logical reason why the OR repertoires of terrestrial and aquatic species differ [5], [17], [80]. The subterranean environment, on the other hand, presents unique challenges. These include the absence of visual cues and limited auditory cues, requiring fossorial species to compensate with enhanced olfaction and hence a diversification in OR genes.

### Purifying Selection and Ancient OR7 Variation

Based on the occurrence of allelic variants of the same OR7 genes, as well as identical OR7 sequences across mole-rat species, we suggest that a proportion of the variability observed in the Bathyergidae might be of ancient origin. This idea is also supported by the clustering of sequences into distinct OR7 lineages, rather than in a species-specific manner (Figure S1). It is worth noting that the majority of functional “ancient” bathyergid OR7 alleles, i.e. conserved alleles from a single OR gene that are present across numerous mole-rat species, occur within clade D (70%) only. This clade was identified as evolving under strong purifying selection and supports the highest number and proportion of functional OR7 genes. Conserved OR7 alleles across this clade may represent allelic variants that maintain precise binding properties among bathyergid species, enabling them to detect primary olfactants e.g. plant exudates released from roots of edible plants [40], [41].

The occurrence of ancient OR loci has also been reported in a comparative study of whole Mouse and Rat OR subgenomes, where the presence of conserved OR loci across species is proposed to be the result of ‘slow OR evolution’ [81]. The authors propose a scenario where the genes have evolved under neutrality, such that loci shared between the two species are a consequence of relatively recent divergence; *Mus* and *Rattus* are estimated to have diverged ∼23 MYA [72]. We argue that purifying selection in the African mole-rats has ensured the persistence of conserved OR loci across all the major generic divergence events in the family i.e. *Bathyergus*, *Georychus*, *Cryptomys* and *Fukomys* onwards (∼17–15 MYA) [66]. Under a scenario of neutral evolution, alleles are predicted to become species-specific only when species have diverged for more than 4Ne generations (where Ne is the effective population size; [82]). Information on bathyergid effective population sizes is not available, but even calculations based on educated estimates suggest highly unrealistic population sizes would be required to account for the retention of conserved OR7 loci due to ‘slow OR evolution’. Conserved OR7 alleles across bathyergid species are therefore most likely the result of strong purifying selection for alleles that confer significant fitness benefits. Indeed, one could envisage that at least some of the conserved OR loci identified by Zhang et al. [81], are a result of selective pressures acting to maintain specific capacities of odour recognition among sympatric Muridae, rather than a by-product of neutral evolution, especially given the divergence time between the two species [72].

### A New Method for Characterising OR Subfamilies

Based on our analyses, the subpool of Bathyergidae OR genes isolated and characterised in this study belongs to a single family of ORs, namely OR7. In order to perform fine-scale classification of the complex vertebrate OR gene superfamily, a number of recent studies subdivide OR families into ‘subfamilies’ based on ‘pattern’ i.e. setting sequence similarity cut-offs of generally 60% [23], [61]. On inspection of average pairwise distances, based on the number of nucleotide differences between functional OR7 genes from clades A–D, we observe that between 62–68% of the sequence similarity occurs across clades. Therefore, if we were to classify Bathyergidae OR7 genes into subfamilies according to ‘pattern’, and using the cut-off limit of 60% [61], the observed clade structure would not reflect subfamily structure. This is because all OR7 genes would fall into a single subfamily. Nevertheless, the results presented here are consistent with the clustering of ORs into clades that have evolved under unrelated selective forces, potentially reflecting their underlying biological significance. Despite the high percentage of between-clade sequence similarity, there appears to be strong functional association between genes belonging to each clade. Thus, it is tempting to speculate that from a functional viewpoint each clade may represent a distinct OR7 subfamily. The above discussion on classification of OR genes into families based on sequence similarity cut-offs, raises the debate of the appropriateness of this practice that has been common in many large-scale OR studies. From our results it is clear that analysis of the evolutionary mechanisms that shape OR genetic diversity across clades can be used as an additional, novel and potentially more accurate method in classifying OR genes, informed by ‘process’ rather than ‘pattern’ alone.

Using a recent dated phylogeny, based on 66 genes and over 2000 mammalian species, the OR7 gene family is thought to have diversified after the Placental-Marsupial split ∼147 MYA [18], [83]. High levels of gene duplication in humans have resulted in OR7 being the largest family of the OR subgenome, occurring as OR7-specific clusters scattered across a number of genomic locations [8]. Although the function of OR7 remains poorly understood, some OR7 genes have played a significant role in recent mammalian evolution, e.g. OR7D4 in humans binds the steroid compounds androstenone and androstadienone [84]. Interestingly, these two compounds were classified as human ‘pheromone candidates’ after they were found to influence both brain function and, more recently, endocrine balance in humans [85]–[87]. These studies suggest that in those species where the VNO is considered to be a ‘nonchemosensory vestige’, like *Homo sapiens* or indeed the naked mole-rat [49], pheromonal communication may still occur, possibly mediated by ORs in the MOE and possibly including loci in the OR7 subfamily.

### Conclusion

This study represents the first assessment of OR7 diversity for a family of subterranean mammals. In exploring the mechanisms shaping the evolution of the African mole-rat olfactory repertoire, we reveal that olfaction in mole-rats has been subject to a spectrum of evolutionary forces. Positive selection emerges as the foremost evolutionary process shaping functional OR7 variability in the family; nonetheless, neither the divergent social strategies of mole-rats nor the specialised subterranean environment emerge as clear drivers of this process. In addition to classic features of ‘birth and death’ evolution [10], an important role for purifying selection also emerges in the evolution of OR7 genes in mole-rats. The ‘clade structure’ observed in the Bathyergidae OR7 gene tree is consistent with a ‘subfamily structure’ based on OR7 functional properties, and likely reflects the broad range of odorant ligands that mole-rat OR7 genes can recognise. These findings challenge the commonly accepted theory that closely related ORs necessarily share functional properties [56], and reveal the intricate mechanisms of OR evolution at a ‘microscopic’ single-OR family scale, thus offering a valuable perspective on the breadth and complexity of OR evolution at the subgenome level.

## Materials and Methods

### Ethics Statement

Mole-rat tissue samples were collected as part of a previous study carried out by Deuve et al. [88]–[90] with full ethics approval by the University of Stellenbosch, Ethics Clearance Certificate # 2006B01006.

### Olfactory Receptor Gene Isolation and Identification

Genomic DNA was extracted from fresh muscle tissue using a standard phenol-chloroform protocol [91]. Species sampled include representative taxa from all currently recognised genera in the Bathyergidae: *Bathyergus janetta* (BJ), *Bathyergus suillus* (BS), *Cryptomys hottentotus hottentotus* (CHH), *Cryptomys hottentotus natalensis* (CHN), *Cryptomys hottentotus pretoriae* (CHP), *Fukomys mechowi* (CM), *Fukomys amatus* (CA), *Fukomys anselli* (CAN), *Fukomys bocagei* (CB), *Fukomys damarensis* (CDM), *Fukomys darlingi* (CD), *Georychus capensis* (GC), *Heliophobius argentocinereus* (HA), *Heterocephalus glaber* (HG).

Vertebrate olfactory receptors display a conserved overall structure typical of GPCRs, with variability concentrated across the ligand binding pockets, spanning transmembrane (TM) domains 3–6 [12], [15]. To provide a reference sequence for the development of bathyergid-specific PCR primers, we used the degenerate PCR primers A4/B6 described in Buck and Axel [4] to amplify TM 2–7 from a single *C. damarensis* individual; conditions followed those reported in Buck and Axel [4]. PCR products were gel purified using the Wizard SV Gel and PCR Clean-up System (Promega) and cloned into *E. coli* DH5α CaCl_2_-competent cells using pGEM-T-Easy Vector System (Promega). Insert-containing clones were sequenced using a BigDye Terminator v.3.1 Cycle Sequencing Kit (Applied Biosystems). Post-sequencing purification was performed using Centrisep Columns (Princeton) and DNA sequences were analysed on an ABI 3130 Genetic Analyser using 3130 Genetic Analyser Data Collection software v.5.2.

Bathyergid-specific OR primers were designed to TM domains 2–7 (approx. size 645 bp): Bathy-OR1 5′- GCG GAC ATC YGT TTC AC - 3′; Bathy-OR2 5′- GTG ACC ACA GTG TAC ATC –3′. The Bathy-OR1/Bathy-OR2 primer pair successfully amplified unambiguous PCR products in all 14 mole-rat species, using the following conditions: 95°C for 1 min, 54°C for 3 min, 72°C for 3 min (35 cycles). Each 40 ul reaction contained between 50–100 ng genomic DNA, 2 pmol/ul of each Bathy-OR1 and Bathy-OR2 primers, 1.5 mM MgCl_2_, 0.2 mM dNTPs, 1.25 U Super-Therm *Taq* DNA polymerase and 1x corresponding *Taq* reaction buffer. PCR products were gel-purified, cloned and sequenced as described previously. Between 10 and 50 clones were sequenced in both direction using using both the forward and reverse-primers for 1 to 3 individuals for each species; this produced 402 OR sequences. Forward and reverse sequences were aligned and checked for ambiguities by eye in Bioedit v7.0.8.0 [92], resulting in 201 putative OR sequences. The sequences were then aligned using Clustal W v2.0 [93], [94] and translated in Bioedit v7.0.8.0 [92]; identical sequences were identified by pairwise comparisons in MEGA v5 [95] and the final data set comprised 178 unique OR sequences.

A BLAST search was performed against the nucleotide collection data, available on NCBI (www.ncbi.nlm.nih.gov), to assign identity to both nucleotide and amino acid OR sequences.

### OR Sequence Identification

The role of recombination in generating sequence variation in any dataset, either *in vivo* or *in vitro*, was evaluated by calculating the level of linkage disequilibrium between polymorphic sites as a function of their physical distance, using Rozas et al’s [96] ZZ value in DnaSP4.5 [97]. This test reduces the possibility of *in vitro* recombination generating false OR variability, since recombination is established as only a minor source of OR variation *in vivo* [10].

Following Steiger et al. [31], OR sequences were classified as pseudogenes if they contained stop codons or frame-shift mutations that disrupted the overall receptor structure. Sequences that translated into putatively functional OR genes, but that differed in length, were considered to be functional only if they maintained the known features of ORs (e.g. the MAYDRFVAIC and KAFSTCASH motifs in TM domains 3 and 6, respectively), and if the variability mapped to the ligand-binding pockets of ORs [14], [15].

In order to identify allelic variants of OR genes, pairwise comparisons were performed across all unique sequences in our dataset using MEGA v5 [95]. Allelic pairs based on the pairwise comparison matrix generated in MEGA were then identified using *alleles.R* (R. Gaujoux, unpublished) developed in R (R Development Core Team, 2008, http://www.R-project.org ). The criteria for allele identification described by Kishida [18] were applied to the bathyergid dataset using the following cut-off limits: within a species, sequences that shared 99% sequence similarity were considered to be alleles of the same gene; across species, the cut-offs were 98% within the same genus and 96% across genera. Single base-pair differences as well as two base-pair differences were assumed to represent identical sequences due to PCR or sequencing errors. Similarly, when two allelic variants shared more than 99% sequence similarity across species (i.e. between 3–5 base pair differences), they were considered to represent identical alleles. When more than two putative alleles of the same OR gene were found in an individual, given the defined cut-offs, two copies of that particular gene were assumed to be present. Similarly, when two presumed alleles were of different functional status i.e. one putatively functional and one pseudogene, they were considered to belong to two different OR genes, the result of a duplication event followed by pseudogenisation. Whenever the percentage sequence similarity led to ambiguous results e.g. when transitivity was not applicable (A = B, B = C but A≠C, with ‘ = ’ meaning ‘alleles’ based on sequence similarity), phylogenetic relationships (described below) were used to allocate alleles to different OR genes. Once identified, alleles of the same OR gene were collapsed down to a single consensus sequence for each putative gene, and used in subsequent analyses.

### Phylogenetic Analyses

Evolutionary relationships among Bathyergidae OR7 genes were explored using maximum likelihood (ML) [98], based on the general time-reversible model (GTR) [99] as determined jModeltest [100], and constructed in MEGA v5 [95]. Tree topology was inferred using all unique mole-rat OR sequences identified, with three non-OR GPCR genes used as an outgroup; robustness of the tree topology was tested using 1000 bootstrap replicates [101]. The resulting tree was used in combination with the pairwise comparison matrix to determine allelic relationships amongst sequences. If sequence similarity led to uncertain allelic allocation, alleles were considered to be sister taxa in the phylogenetic tree. A further ML tree was then constructed (GTR, 1000 bootstrap) using only a single representative sequence for each putative OR gene.

In the most comprehensive survey of mammalian ORs to date, Hayden et al. [22] used a combination of sequence similarity and phylogenetic criteria for OR gene classification. Their dataset analysed the entire OR subgenomes of 50 mammalian species, consisting of ∼50,000 OR sequences. One or two representative sequences per species, for each of the 17 OR families from Hayden et al. [22], were aligned together with the bathyergid OR dataset, using the online Clustal W alignment tool from the European Bioinformatics Institute (available at www.ebi.ac.uk). Aligned sequences were then imported into Bioedit v7.0.8.0 [92] and corrections to the alignment were made by eye. An ML tree was then constructed using the Tamura-Nei substitution model [102] in MEGA v5 [95] with 1000 bootstrap replications. Phylogenetic analysis based on all nucleotide sites included 312 representative sequences from all OR gene families across 18 different mammalian species, as well as the 119 Bathyergidae ORs. All Bathyergidae sequences clustered together with known Family 7 ORs [22]. Family information was included in Bathyergidae sequence nomenclature (GenBank accession numbers KF453235–KF453412).

All the available OR sequences belonging to Family 7 from Hayden et al’s dataset [22], representing the entire Family 7 OR subgenome of 18 different mammalian species, were then aligned with the 119 mole-rat sequences using the online Clustal W tool. Aligned sequences were corrected by eye in Bioedit v7.0.8.0 [92], and a final ML tree (Tamura-Nei) was constructed in MEGA v5 and tested using 1000 bootstraps replicates. Positions containing alignment gaps were eliminated from the pairwise sequence comparisons (pairwise deletion option), resulting in 805 nucleotide positions in the final dataset.

### Signatures of Selection on Bathyergid OR7 Genes

Because olfactory receptors display a highly conserved overall structure, with variability limited to a set of amino acid residues involved in the binding of odorant molecules, an average of substitution rates across the entire OR gene provides neither an accurate nor informative test of positive selection [12], [15]. Therefore, tests for positive selection were applied to different codon sites in the dataset using the SELECTON server (available at http://selecton.tau.ac.il/) [103], [104]. Estimates of the ratio of non-synonymous (dN) to synonymous (dS) substitutions were obtained for each codon, and significance assessed via a LRT. The LRT compared two nested models for each codon: a null model (M8a), which assumes no selection, and an alternative model (M8) which allows positive selection to occur. Three sets of bathyergid OR7 genes, corresponding to clades A, C and D, identified in the phylogenetic analysis, were analysed separately using codon-based multiple sequence alignment (MSA); pseudogenes were not included in the analysis. Clade B displayed only three putatively functional OR genes, and was therefore excluded from this analysis due to insufficient sample size. Across clade D, several codons with dN/dS ratios <1 were identified, while none appeared to have dN/dS >1. A codon-based-Z test, to test for purifying selection (overall average), was performed on clade D’s functional ORs using the Nei-Gojobori method [105] implemented in MEGA5 [95], with 1000 bootstrap replicates to determine significance of purifying selection across this clade.

To investigate whether positive selection may have acted along specific bathyergid OR7 lineages, a branch-site test (test 2 in [106]) was carried out in PAML [107], [108] using CodeML and based on the ML tree of African mole-rat OR7 genes. CodeML was used to estimate the dN/dS ratio (ω) on codon (nucleotide) alignments across the topology of the trees. Two nested models, null and alternative, were computed and compared using a LRT. In the null model, codons along all branches are either under purifying selection (ω <1) or under neutral evolution (ω = 1), and the foreground branch may have different proportions of sites under neutral selection than the background branches (i.e. relaxed purifying selection). In the alternative model, some sites on the foreground branch may be under positive selection (ω >1). Following Yang [109], stop codons and alignment gaps were excluded from the alignment used to construct a ML tree (as previously described), and the resulting tree maintained the same tree topology as the original bathyergid OR tree. In the branch-site test of positive selection, each branch of the OR gene tree was labelled in turn as foreground; a LRT was performed on all pairs of nested models and compared to a χ^2^ distribution to determine significance. Furthermore, the Q-value, a measure of the false discovery rate (FDR) due to multiple testing, was calculated for each branch using the ‘Q-value’ software available at http://genomics.princeton.edu [110]–[112]. When the LRT remained significant after the correction for multiple testing (i.e. both p- and Q-values<0.05), the posterior probability of sites being under positive selection (dN/dS >1) was calculated using the BEB method [64], [65] implemented in CodeML.

### Testing the Role of Sociality in OR7 Variation

To test the role of sociality in shaping OR7 variation, we partitioned the OR7 gene tree between Solitary and Social bathyergid species (following Ramm et al. [67]), and performed a branch-site test of positive selection as previously described. The tree was partitioned by labelling the terminal branches of the phylogeny according to the social status of the corresponding species. The analysed data comprised all 119 unique bathyergid OR genes identified in this study, including both functional OR7 genes and pseudogenes. Stop codons from OR pseudogenes and alignment gaps were excluded from the OR7 alignment. OR7 genes from the genera *Bathyergus*, *Georychus* and *Heliophobius* were labelled as Solitary, while those belonging to *Cryptomys, Fukomys* and *Heterocephalus* were labelled Social [33].

We did not hypothesise *a priori* which social system (i.e. solitary or social) would be subject to positive selection, and therefore conducted two branch-site analyses. In the first analysis we tested whether social lineages carried a signal of increased selection when compared to the solitary ones, labelling all the terminal branches of the OR7 gene tree that belonged to social bathyergid as ‘foreground’. In the second analysis, the test was performed with the ‘solitary leaves’ of the tree labelled as foreground. With these branches defined as foreground, a LRT was performed on all pairs of nested models (null and alternative) and compared to a χ^2^ distribution to determine significance. A Q-value was then calculated for each branch using the ‘Q-value’ software as before. When the LRT was significant with a FDR below 5%, the posterior probability of sites being under positive selection (dN/dS >1) was then calculated using the BEB method in CodeML.

### The Subterranean Ecogroup as a Driver of Bathyergid OR7 Diversification

The role of the subterranean environment as a driver of OR evolution across the Bathyergidae, was explored by comparing the ratios of functional OR genes:pseudogenes across ecotypes (following [22]). Hayden et al.’s dataset [22], comprising ratios of OR functional genes:pseudogenes from whole-OR-subgenome data for 50 mammalian species, covered a range of environmental niches, namely: Terrestrial, Aquatic, Semi-aquatic and Volant (i.e. bats). Hayden et al. [22] performed a Bayesian phylogenetic analysis to classify OR genes into gene families, and the 17 ‘traditional’ OR families were recovered [61]; the following families were found to group together; OR 2/13; OR 1/3/7; OR 5/8/9. Data for this part of the study is available at http://genome.cshlp.org/content/20/1/1/suppl/DC1.

The authors used a principal component analysis, based on the different proportions of functional ORs and pseudogenes across gene families, to then compare data from the different ecogroups, and in so doing identify the OR families that explain most of the variation between these groups.

Following Hayden et al. [22], OR7 belongs to the broader mammalian OR grouping of OR 1/3/7. Here, we used the numbers of functional OR 1/3/7 genes and pseudogenes, as well as their relative proportions as reported in [22], together with data from the 14 Bathyergidae species analysed in this study, to test our hypothesis that the subterranean niche has contributed to the diversification of functional variability in the mole-rat OR genome. In this context, species used in the analysis were classified according to five ecogroups: Aquatic, Semi-aquatic, *Subterranean*, Terrestrial and Volant.

OR1/3/7 ratios were plotted across all species within each ecogroup, and the mean percentage of pseudogenes and associated standard error were calculated in R (R Development Core Team, 2008, http://www.R-project.org). To test for pairwise differences in the distributions of pseudogene proportions between each ecogroup, we applied a non-parametric Wilcoxon-test [113], as well as the Benjamini & Hochberg ‘BH’ correction for multiple testing [114].

## Supporting Information

Figure S1 **Bathyergid OR7 gene tree.** Maximum-likelihood tree (GTR, 1000 bootstrap) constructed using all 178 unique Bathyergid OR sequences; three rhodopsin-like GPCRs are used as outgroups (accession numbers NP_001287.2, NP_005292.2, NP_037014.2). The four main OR clades are indicated (A–D); only one isolated gene (BJ4_A12) falls out of these clades and is labelled with an asterisk. Abbreviations correspond to gene names in Genbank accession numbers KF453235–KF453412 and contain species information as follows: *Bathyergus janetta* (BJ), *Bathyergus suillus* (BS), *Cryptomys hottentotus hottentotus* (CHH), *Cryptomys hottentotus natalensis* (CHN), *Cryptomys hottentotus pretoriae* (CHP), *Fukomys mechowi* (CM), *Fukomys amatus* (CA), *Fukomys anselli* (CAN), *Fukomys bocagei* (CB), *Fukomys damarensis* (CDM), *Fukomys darlingi* (CD), *Georychus capensis* (GC), *Heliophobius argentocinereus* (HA), *Heterocephalus glaber* (HG).
(TIF)

Figure S2 **Mammalian OR7 gene tree.** Maximum likelihood tree (Tamura-Nei, 1000 boostrap) constructed with all the available mammalian OR7 genes [22]. Each circle dot corresponds to an OR7 gene belonging to family 7; ORs from different taxonomic families are colour-coded as indicated on the figure. Rhodopsin-like non-OR GPCRs are used as an outgroup (accession numbers NP_001287.2, NP_005292.2, NP_037014.2). Bathyergidae ORs from clades A–D are indicated in green; bootstrap values are reported for the main bathyergid clades.
(TIF)

Table S1 **Numbers of functional ORs and pseudogenes in clades A–D.**
(DOCX)

Table S2 **Positively selected residues in Bathyergidae OR7 lineages.**
(DOCX)
