# Supplementary figures and images for: Genetic Signatures for Enhanced Olfaction in the African Mole-Rats
Source: PLoS One. 2014 Apr 3;9(4):e93336. doi: 10.1371/journal.pone.0093336 (PMC3974769; doi:10.1371/journal.pone.0093336)

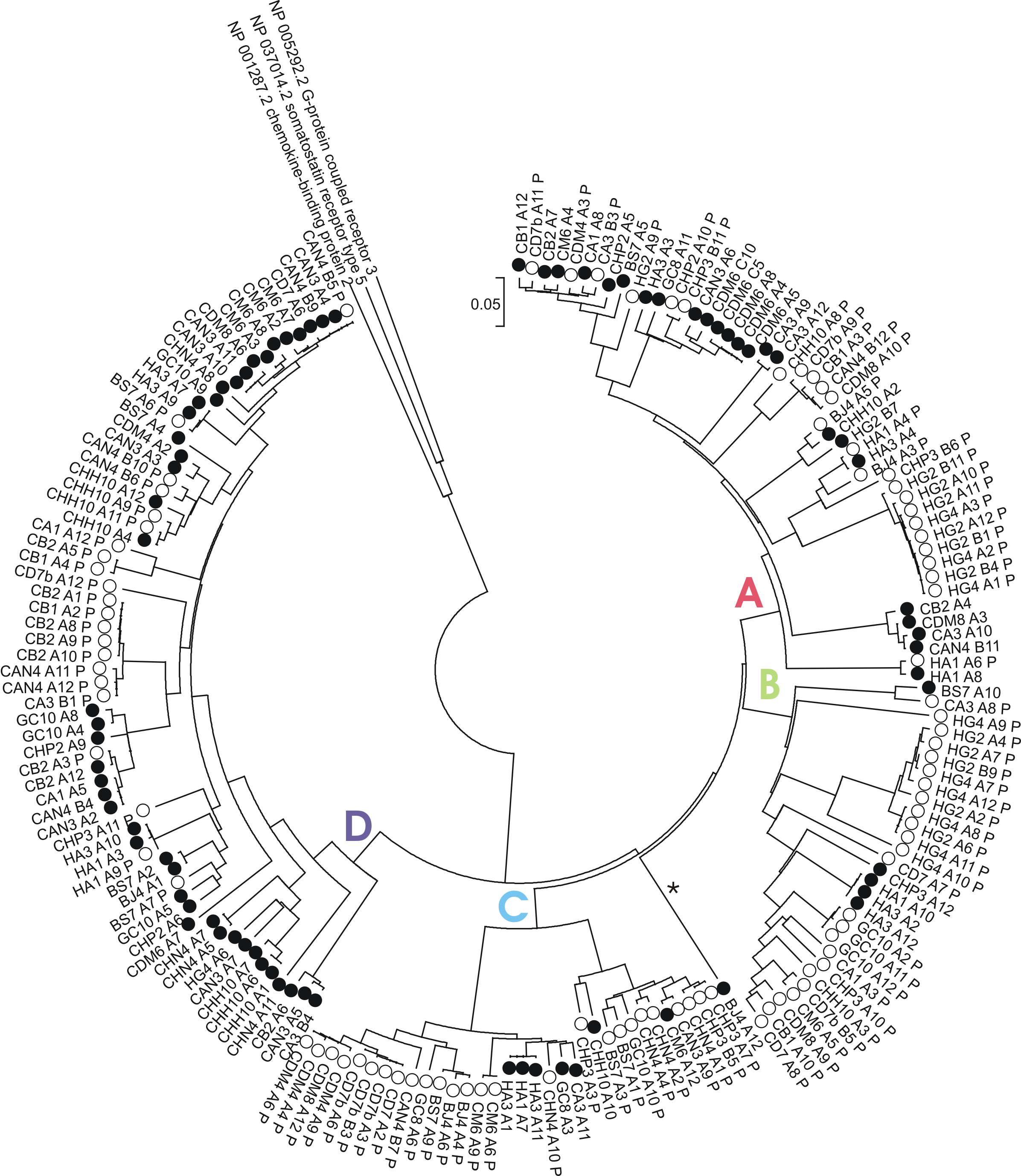

Supplement: Figure S1 — Bathyergid OR7 gene tree. Maximum-likelihood tree (GTR, 1000 bootstrap) constructed using all 178 unique Bathyergid OR sequences; three rhodopsin-like GPCRs are used as outgroups (accession numbers NP_001287.2, NP_005292.2, NP_037014.2). The four main OR clades are indicated (A–D); only one isolated gene (BJ4_A12) falls out of these clades and is labelled with an asterisk. Abbreviations correspond to gene names in Genbank accession numbers KF453235–KF453412 and contain species information as follows: Bathyergus janetta (BJ), Bathyergus suillus (BS), Cryptomys hottentotus hottentotus (CHH), Cryptomys hottentotus natalensis (CHN), Cryptomys hottentotus pretoriae (CHP), Fukomys mechowi (CM), Fukomys amatus (CA), Fukomys anselli (CAN), Fukomys bocagei (CB), Fukomys damarensis (CDM), Fukomys darlingi (CD), Georychus capensis (GC), Heliophobius argentocinereus (HA), Heterocephalus glaber (HG). (TIF) [file pone.0093336.s001.tif]

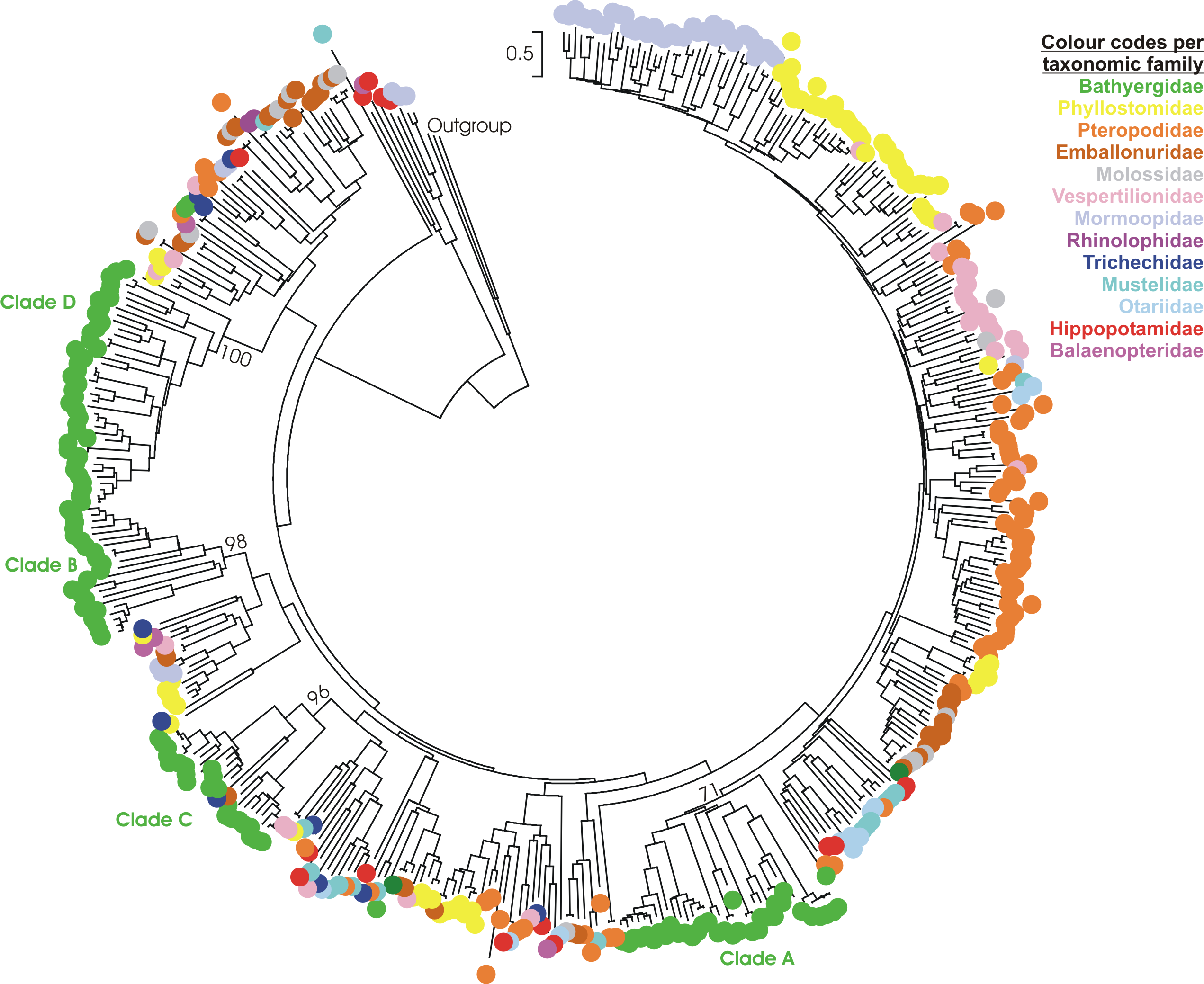

Supplement: Figure S2 — Mammalian OR7 gene tree. Maximum likelihood tree (Tamura-Nei, 1000 boostrap) constructed with all the available mammalian OR7 genes [22]. Each circle dot corresponds to an OR7 gene belonging to family 7; ORs from different taxonomic families are colour-coded as indicated on the figure. Rhodopsin-like non-OR GPCRs are used as an outgroup (accession numbers NP_001287.2, NP_005292.2, NP_037014.2). Bathyergidae ORs from clades A–D are indicated in green; bootstrap values are reported for the main bathyergid clades. (TIF) [file pone.0093336.s002.tif]
