# Supplementary material for: Genetic Signatures for Enhanced Olfaction in the African Mole-Rats
Source: PLoS One. 2014 Apr 3;9(4):e93336. doi: 10.1371/journal.pone.0093336 (PMC3974769; doi:10.1371/journal.pone.0093336)
Supplement: Table S1 — Numbers of functional ORs and pseudogenes in clades A–D. (DOCX) [file pone.0093336.s003.docx]

| **Clade** | **Functional ORs** | **Pseudo-ORs** | **% Functional ORs** | **% Pseudo- ORs** |
| --- | --- | --- | --- | --- |
| A | 17 | 17 | 50 | 50 |
| B | 3 | 18 | 14 | 86 |
| C | 5 | 17 | 23 | 77 |
| D | 25 | 15 | 63 | 37 |
