# Supplementary material for: Genetic Signatures for Enhanced Olfaction in the African Mole-Rats
Source: PLoS One. 2014 Apr 3;9(4):e93336. doi: 10.1371/journal.pone.0093336 (PMC3974769; doi:10.1371/journal.pone.0093336)
Supplement: Table S2 — Positively selected residues in Bathyergidae OR7 lineages. (DOCX) [file pone.0093336.s004.docx]

| **Clade** | **Branch** | **Amino-acid position ♦** | **BEB*** | **Location domain ♦** |
| --- | --- | --- | --- | --- |
| **A** | 34 | 30 | 0.744 | TM3 |
|  |  | 99 | 0.754 | EC2 |
|  |  | 102 | 0.809 | EC2 |
|  |  | 158 | 0.778 | IC3 |
|  |  | 162 | 0.912 | IC3 |
|  |  | 186 | 0.983 | TM6 |
|  |  | 202 | 0.818 | EC3 |
|  |  | 205 | 0.657 | TM7 |
| **A** | 27 | 7 | 0.993 | TM2 |
| **C** | 63 | 20 | 0.595 | EC1 |
|  |  | 177 | 0.542 | TM6 |
|  |  | 179 | 0.843 | TM6 |
| **C** | 75 | 7 | 0.649 | TM2 |
|  |  | 21 | 0.882 | EC1 |
|  |  | 37 | 0.942 | TM3 |
|  |  | 40 | 0.86 | TM3 |
|  |  | 41 | 0.807 | TM3 |
|  |  | 46 | 0.829 | TM3 |
|  |  | 47 | 0.805 | TM3 |
|  |  | 48 | 0.775 | TM3 |
|  |  | 49 | 0.851 | TM3 |
| * Results from the Bayes Empirical Bayes (BEB) analysis indicate the probability of the corresponding amino-acid residues being under positive selection. Branch numbers match labelled branches in Figure 3. | | | | |
| ♦ Amino-acid positions and location domains were assigned based on the molecular model represented in Figure 1. | | | | |
